# Supplementary material for: Association of Dietary Nitrate, Nitrite, and N-Nitroso Compounds Intake and Gastrointestinal Cancers: A Systematic Review and Meta-Analysis
Source: Toxics. 2023 Feb 17;11(2):190. doi: 10.3390/toxics11020190 (PMC9962651; doi:10.3390/toxics11020190)
Supplement: Supplementary file 1 [file toxics-11-00190-s001.zip › toxics-2092528-Supplementary Table S2.pdf]

Supplementary Table S2

Table S2: Characteristics of case-control studies in the systematic review.

num	Firstauthor_ Year_Gender_Locatio	Number of Cases	Numberof Controls	Cancer type	Analytical Category	Consumption Categories	Adjusted OR 95CI	adjusted
**1**	Angela Coss_2003_ F_USA_Animal sources	32	164	Pancrase__	Nitrite_ mg/day	0.13-0.18	2.4(1.2-4.7)	reference dose<0.13- adjusted: age, cigarette use, energy
		26	147			0.19-0.26	1.9(0.94-4.0)	
		51	180			>0.26	3.2(1.6-6.4)	
	Angela Coss_2003_ M_USA_Animal sources	22	282			0.22-0.31	2.1(0.95-4.8)	reference dose<0.22- adjusted: age, cigarette use, energy
		60	359			0.32-0.53	3.8(1.8-8.0)	
		50	342			>0.53	2.3(1.1-5.1)	
	Angela Coss_2003_ F_USA_Food	32	146		Nitrite_ mg/day	0.56-0.71	1.8(0.94-3.4)	reference dose<0.56- adjusted: age, cigarette use, energy
		32	168			0.72-0.93	1.4(0.72-2.6)	
		40	181			>0.93	1.3(0.65-2.5)	
		33	157		Nitrate_ mg/day	63-90	0.99(0.58-1.7)	reference dose<63- adjusted: age, cigarette use, energy
		24	158			91-126	0.64(0.36-1.1)	
		26	160			>126	0.53(0.29-0.97)	
	Angela Coss_2003_ M_USA_Food	22	307		Nitrite_ mg/day	0.75-0.98	1(0.52-2.0)	reference dose<0.75- adjusted: age, cigarette use, energy
		40	333			0.99-1.30	1.5(0.81-2.9)	
		64	374			>1.30	1.5(0.79-3.0)	
		33	311		Nitrate_ mg/day	58-82	1.1(0.63-1.9)	reference dose<58- adjusted: age, cigarette use, energy
		39	311			83-117	1.2(0.70-2.0)	
		43	327			>117	1(0.60-1.8)	
**2**	Buiatti, E._1990_ M/F_Italy_Food	203	231	Stomach__	Nitrate_ mg/day	81	0.9(0.7-1.1)	reference dose:53- adjusted: non-dietary variables and kilocalorie
						103	0.9(0.6-1.1)	
						130	0.7(0.5-0.9)	
						193	0.9(0.7-1.2)	
					Nitrite_ mg/day	2.8	1(0.8-1.4)	reference dose:2.1- adjusted: non-dietary variables and kilocalorie
						3.4	1.2(0.9-1.7)	
						4.1	1.4(1.0-2.0)	
						5.9	1.9(1.3-2.7)	
**3**	De Roos, A. J._2003_ M/F__Water	116	380	Colon__	Nitrate_ mg/l	>1 to <=3	1(0.8-1.3)	reference dose<1- adjusted: the frequency-matched factors of age and sex
		27	124			>3 to <=5	0.7(0.4-1.1)	
		61	174			>5	1.2(0.8-1.7)	
		98	380	Rectum__		>1 to <=3	0.8(0.6-1.1)	
		30	124			>3 to <=5	0.7(0.5-1.2)	
		56	174			>5	1.2(0.8-1.8)	
**4**	De Stefani, E._1998_ M/F_Uruguay_Food	340	698	Stomach__	NDMA_		1.51(1.33-1.72)	Adjusted: age, sex, and smoking. alcohol, residence

	De Stefani, E._1998_ M_Uruguay_Food	224	459				1.63(1.39-1.91)	
	De Stefani, E._1998_ F_Uruguay_Food	116	239				1.34(1.08-1.67)	
	De Stefani, E._1998_ F_Uruguay_Food	116	239		Nitrite_		0.79(0.62-1.01)	
	De Stefani, E._1998_ M_Uruguay_Food	224	459				0.52(0.43-0.62)	
	De Stefani, E._1998_ M/F_Uruguay_Food	340	698				0.55(0.48-0.62)	
**5**	De Stefani, E._2001_ M/F_Uruguay_Animal sources	27	88	Stomach__	NDMA_ micro/day	2.0-2.5	2.2(1.2-4.1)	reference dose<=1.9- adjusted: age, gender, residence, education
		57				>2.6	2.2(1.2-4.1)	
		41			Nitrate_ mg/day	524-784	1.1(0.6-1.8)	reference dose<523- adjusted: age, gender, residence, education
		34				>785	0.9(0.5-1.6)	
		48			Nitrite_ mg/day	6.3-10.0	1.5(0.9-2.6)	reference dose<6.2- adjusted: age, gender, residence, education
		43				>10.1	1.8(1.0-3.2)	
**6**	Espejo-Herrera, N._ 2016_F_spain and italy_Water	158	349	Colorectal_	Nitrate_ mg/day	>5-10	1.2(0.90-1.58)	reference dose<5- adjusted: sex, age, education, BMI, physical activity, NSAI drug , family history, intake of energy, oral contraceptives use
		247	360			>10	1.41(1.04-1.91)	
		30	349	Rectum__		>5-10	0.87(0.52-1.45)	
		70	360			>10	1.49(0.89-2.48)	
		122	349	Colon__		>5-10	1.33(0.97-1.80)	
		174	360			>10	1.46(1.04-2.05)	
	Espejo-Herrera, N._ 2016_M_spain and italy_Water	289	454	Colorectal_		>5-10	1.16(0.94-1.44)	
		397	468			>10	1.5(1.21-1.87)	
		80	454	Rectum__		>5-10	0.94(0.68-1.28)	
		133	468			>10	1.55(1.16-2.08)	
		202	454	Colon__		>5-10	1.26(0.99-1.61)	
		260	468			>10	1.51(1.17-1.94)	
	Espejo-Herrera, N._ 2016_M/F_spain and italy_Water	447	803	Colorectal_		>5-10	1.17(0.98-1.38)	
		644	828			>10	1.49(1.24-1.78)	
		110	803	Rectum__		>5-10	0.93(0.70-1.23)	
		203	828			>10	1.62(1.23-2.14)	
		324	803	Colon__		>5-10	1.28(1.06-1.55)	
		434	828			>10	1.52(1.24-1.86)	
	Espejo-Herrera, N._ 2016_M/F_spain and italy_Food	564	1058	Colorectal_		83-133	0.97(0.83-1.14)	reference dose: <83 - adjusted: sex, age, education, physical activity, NSAI drug, family history, BMI, intake energy, fiber
		527	1057			>133	0.84(0.70-1.00)	
		394	1058	Colon__		83-133	1.04(0.87-1.24)	
		371	1057			>133	0.9(0.74-1.10)	
		161	1058	Rectum__		83-133	0.85(0.66-1.08)	
		151	1057			>133	0.76(0.58-1.00)	
		578	1058	Colorectal_		4.5-6.8	1.15(0.98-1.35)	

	Espejo-Herrera, N._2016_M/F_spain and italy_Animal sources	634	1057			>6.8	1.16(0.98-1.38)	reference dose: <4.5 - adjusted: sex, age, education, physical activity, NSAI drug, family history, BMI, intake energy
		191	1058	Rectum__		4.5-6.8	1.59(1.22-2.06)	
		204	1057			>6.8	1.55(1.17-2.05)	
		423	1057	Colon__		>6.8	1.06(0.87-1.30)	
		378	1058			4.5-6.8	1.03(0.86-1.24)	
	Espejo-Herrera, N._2016_M/F_spain and italy_Plant sources	575	1058	Colorectal_		68-118	0.99(0.85-1.16)	reference dose: <68 - adjusted: sex, age, education, physical activity, NSAI drug, family history, BMI, intake energy, fiber
		513	1057			>118	0.83(0.70-0.99)	
		169	1058	Rectum__		68-118	1.04(0.71-1.16)	
		144	1057			>118	0.9(0.57-0.99)	
		397	1058	Colon__		68-118	1.04(0.87-1.24)	
		364	1057			>118	0.89(0.73-1.08)	
**7**	Fathmawati_2017_M/F_Indonesia_Water	3	4	Colorectal_	Nitrate_ mg/l	>50	1.405(0.14-13.67)	adjusted: protein intake, smoking history, age, family history of cancer, diabetic
		16	4			>50	4.312(1.31-14.09)	
**8**	Hernández-Ramírez, R. U._2009_M/F_Mexico_Food	82	159	Stomach__	Nitrite_ mg/day	>1.0-1.2	1.07(0.69-1.65)	reference dose<1.0- adjusted: energy, age, gender
		82	159			>1.2	1.52(0.99-2.34)	
		76	156		Nitrate_ mg/day	>90.4-141.7	0.93(0.62-1.39)	reference dose: <90.4- adjusted: energy, age, gender
		76	156			>141.7	0.61(0.39-0.96)	
		82	159		Nitrite_ mg/day	>0.2-0.4	0.78(0.50-1.21)	reference dose<0.2- adjusted: energy, age, gender
		82	159			>0.4	1.56(1.02-2.4)	
		76	156		Nitrate_ mg/day	>1.7-3.9	1.28(0.82-2.0)	reference dose: <1.7- adjusted adj: energy, age, gender
		76	156			>3.9	1.92(1.23-3.02)	
		82	159		Nitrite_ mg/day	>0.1-0.2	0.81(0.54-1.21)	reference dose<0.1- adjusted: energy, age, gender
		82	159			>0.2	0.77(0.50-1.18)	
		76	156		Nitrate_ mg/day	>81.7-134.9	0.93(0.62-1.39)	reference dose: <81.7- adjusted: energy, age, gender
		76	156			>134.9	0.62(0.40-0.97)	
**9**	Jakszyn, P._2006_M/F_European countries_Food	105	146359	Stomach__	NDMA_ micro/day	.09	0.87(0.64–1.2)	reference dose: <0.09- adj:sex,height,weight,education,smoking,physical activity, fruits intake, energy, nitrites
		31	146359			.195	0.99(0.69–1.41)	
		31	146359	Stomach_ Noncardia		.09	1.04(0.66–1.63)	
		52	146359			.195	1.09(0.65–1.81)	
		52	146359	Stomach_ Cardia_		.09	0.74(0.41–1.34)	
		105	146359			.195	0.68(0.34–1.37)	
**10**	Kim, H. J._2007_ M/F_Korea_Food	67	68	Stomach__	Nitrate_ mg/day	458	1.13(0.54–2.36)	reference dose: 240- adjusted: age (<50, 50–59, 60–69, and ≥70 yr), sex, socioeconomic status (low, medium, and high status; low status indicates below elementary school in education and $ 8500 in annual income), family history (yes and no for only first-degree relatives), refrigerator use (<20 yr and ≥20 yr), H. pylori infection, and foods (charcoal grilled beef, Korean cabbage kimchi, Dongchimi, spinach,
		32	34			811	1.13(0.42–3.06)	

								garlic, mushroom, and salty foods; low, medium, and high intake).
**11**	La Vecchia, C._1994_ M/F_Italy_Food	128	404	Stomach__	Nitrite_ mg/day	2.41	0.98(0.72-1.33)	refrence:1.91- adjusted: multiple logistic regression(age, sex, education, family history, BMI, total energy)
		126	405			2.94	0.99(0.72-1.36)	
		153	406			3.64	1.15(0.84-1.59)	
		193	404			>3.64	1.35(0.96-1.88)	
		156	405		Nitrate_ mg/day	80.7	0.64(0.49-0.83)	reference:62.96- adjusted: multiple logistic regression (age, sex, education, family history, BMI, total energy)
		117	404			96.33	0.5(0.38-0.67)	
		117	406			116.88	0.52(0.39-0.70)	
		105	404			>116.88	0.43(0.32-0.59)	
**12**	La Vecchia, C._1995_ M/F_Italy_Food	231	687	Stomach__	NDMA_ micro/day	0.131-0.190	1.11(0.9-1.4)	Reference dose<=0.130- adjusted: age, sex, education, family history. carotene, vitamin C, total calories, nitrite, nitrate
		308	683			>0.191	0.191(1.1-1.7)	
**13**	La Vecchia, C._1997_ M/F_Italy_Food	407	987	Stomach__Carcinoma	Nitrite_ mg/day	>=2.7	1.44(1.2-1.7)	reference dose<2.7-adj:sex,age,education,
**14**	Lopez-Carrillo, L._2004_ M/F_Mexico_Food	60	146	Stomach__	Nitrite_ portions/day	0.12–0.26	0.95(0.62-1.46)	reference dose=0-0.11- adjusted: age, gender, energy, socioeconomic ,education, Hp/CagA status and ascorbic acid
		83	148			0.27–2.25	1.24(0.81-1.90)	
**15**	Mayne, S. T._2001_ M/F_USA_Food	255	687	Stomach_ Cardia_Adenocarcinoma	Nitrite_ mg/day		1.12(0.87-1.44)	adjusted: sex; site (Connecticut, Washington, New Jersey); age; race (white versus other); proxy status; income; education; usual body mass index; cigarettes/day; years of consuming beer, wine, and liquor (each); and energy intake.
		352	687	Stomach_Non cardia _Adenocarcinoma			1.64(1.30-2.07)	
		206	687	Esophagus__Squamous cell carcinoma			1.12(0.84-1.51)	
		282	687	Esophagus__Adenocarcinoma			1.02(0.80-1.30)	
**16**	McElroy, J. A._2008_ M/F_USA_Water	22	19	Colorectal_	Nitrate_ mg/l	0.5-1.9	1.39(1.02-1.89)	reference dose<0.5- adjusted: age, interview period
		29	25			2.5-5.9	1.32(0.99-1.76)	
		12	11			6.0-9.9	1.28(0.88-1.88)	
		7	6			>10.0	1.57(0.97-2.52)	
		23	19	Rectum__		0.5-1.9	1.29(0.73-2.31)	
		28	25			2.5-5.9	1.19(0.69-2.06)	
		10	11			6.0-9.9	1.11(0.52-2.36)	
		5	6			>10.0	1.26(0.47-3.43)	
		24	19	Colon_ distal _		0.5-1.9	1.58(1.03-2.40)	
		29	25			2.5-5.9	1.38(0.92-2.06)	
		12	11			6.0-9.9	1.43(0.85-2.41)	

		5	6			>10.0	1.23(0.59-2.56)	
		20	19	Colon_ Proximal_		0.5-1.9	1.35(0.81-2.26)	
		28	25			2.5-5.9	1.36(0.85-2.17)	
		12	11			6.0-9.9	1.34(0.73-2.47)	
		11	6			>10.0	2.76(1.42-5.38)	
**17**	Miller, P. E._2013_ M/F_USA_Animal sources	177	207	Colorectal_	Nitrate and Nitrite_ micro/1000 kcal	114.6-197.0	0.98(0.72-1.32)	reference dose< 114.6 - adjusted: age, sex, total energy intake, body mass index, past regular NSAID use, and fruit and vegetable consumption
		194	207			197.1-310.2	1.07(0.79-1.45)	
		211	207			310.3-496.6	1.09(0.80-1.47)	
		225	207			> 496.6	1.19(0.87-1.61)	
		124	207	Colon__		114.6-197.0	1.02(0.73-1.42)	
		140	207			197.1-310.2	1.15(0.83-1.61)	
		146	207			310.3-496.6	1.14(0.82-1.60)	
		157	207			> 496.6	1.28(0.92-1.80)	
		75	207	Colon_ Proximal_		114.6-197.0	1.05(0.71-1.56)	
		86	207			197.1-310.2	1.25(0.85-1.86)	
		76	207			310.3-496.6	1.06(0.71-1.58)	
		102	207			> 496.6	1.57(1.06-2.34)	
		45	207	Colon_ distal_		114.6-197.0	0.99(0.62-1.59)	
		50	207			197.1-310.2	1.06(0.67-1.70)	
		64	207			310.3-496.6	1.28(0.81-2.01)	
		51	207			> 496.6	0.98(0.61-1.58)	
		52	207	Rectum__		114.6-197.0	0.95(0.61-1.48)	
		54	207			197.1-310.2	0.96(0.62-1.50)	
		63	207			310.3-496.6	1.02(0.66-1.58)	
		67	207			> 496.6	1.04(0.67-1.62)	
**18**	Palli, D._2001_ M/F_Italy_Food	286	187	Stomach__	NDMA_ micro/day	.2	1.1(0.8-1.6)	reference dose: 0.12- adjusted: multiple logistic regressions(age, sex, social class, family history, BMI, total energy)
		286	187			.33	1.1(0.8-1.5)	
		286	187		Nitrate_ mg/day	93.2	0.7(0.5-1.0)	reference dose: 62.6- adjusted: multiple logistic regressions(age, sex, social class, family history, BMI, total energy)
		286	187			132.9	0.6(0.4-0.9)	
		286	187		Nitrite_ mg/day	3.5	1.4(1.0-2.0)	reverence dose: 2.5- adjusted: multiple logistic regressions(age, sex, social class, family history, BMI, total energy)
		286	187			5.4	1.4(1.0-2.0)	
**19**	Pobel, D._1995_ M/F_France_Food	31	43	Stomach__	NDMA_ micro/day	.25	4.13(0.93-18.27)	reference dose:0.20- adjusted: age, sex, occupation , calorie intake
						.51	7(1.85-26.46)	
					Nitrite_ mg/day	1.98	0.83(0.41-1.67)	reverence dose:1.61- adjusted: age, sex, occupation , calorie intake
						2.26	0.88(0.44-1.79)	
					Nitrate_ mg/day	137.26	0.49(0.24-1.01)	reverence dose:89.03- adjusted: age, sex, occupation , calorie intake
						192.73	0.76(0.38-1.50)	
	Pobel, D._1995_				Nitrite_	1.98	0.74(0.37-1.48)	

	M/F_France_Plant sources				mg/day	2.26	0.77(0.38-1.57)	reverence dose:1.61- adjusted: age, sex, occupation , calorie intake
					Nitrate_ mg/day	137.26	0.52(0.26-1.07)	reverence dose:89.03- adjusted: age, sex, occupation , calorie intake
						192.73	0.73(0.37-1.45)	
**20**	Rick J.Jansen_2013_ M/F_USA_Animal sources	79	197	Pancrase__	Nitrate_ mg/day	.03	0.57(0.40-0.83)	reference dose:Q1:0.01-adjusted: cigarette smoking, pack-years, pack-years squared
		72	197			.06	0.48(0.33-0.70)	
		60	196			.12	0.38(0.26-0.57)	
		48	196			.26	0.26(0.17-0.40)	
**21**	Rogers, MA._1995_ M/F_USA_Food	23	147	Esophagus	NDMA_ micro/day	0.06-0.179	1.31(0.60-2.85)	Reference dose<=0.06- adjusted: age, gender, pack-years of cigarettes .drink alcohol. Energy intake .ascorbic acid intake, body mass index. Level of education
		52	147			>0.179	1.86(0.87-3.95)	
		28	134		Nitrite_ mg/day	1.06-1.60	1.17(0.57-2.38)	Reference dose: <1.06 - adjusted: age, gender, pack-years of cigarettes .drink alcohol. Energy intake. Ascorbic acid intake, body mass index. Level of education
		43	151			>1.60	1.58(0.73-3.44)	
		39	144		Nitrate_ mg/day	134-226	0.71(0.38-1.33)	Reference dose: <134 - adjusted: age, gender, pack-years of cigarettes .drink alcohol. Energy intake. Ascorbic acid intake, body mass index. Level of education
		25	140			>226	0.44(0.24-0.93)	
**22**	Taneja,P._2017_M/F_India_Water	41	24	Stomach__	Nitrate_ mg/l	<=45	1(0.98-1.01)	adjusted: age, gender, tobacco
		37	132			>45	1.1(0.99-1.15)	
**23**	Ward, M. H._2007_ M/F_maryland_Animal sources	27	57	Colorectal_	Nitrite_ mg/day	0.02 - 0.07	1(0.7-2.3)	adjusted: age, gender, calories, smoking
		39	57			0.08 to <0.16	1.2(0.9-3.2)	
		52	57			0.16-1.23	1.7(0.8-3.5)	
		30	57		Nitrate and Nitrite_ mg/day	0.22 to <0.89	1.1(0.6-2.5)	reference dose:0 to<0.22- adjusted: age, gender, calories, smoking
		34	57			0.89 to <1.86	1.3(1.0-3.9)	
		57	57			1.86-12.28	2(1.0-1.8)	
**24**	Ward, M. H._2008_ M/F_USA_Animal sources	17	99	Esophagus__Adenocarcinoma	Nitrate and Nitrite_ mg/day	3.8-<5.7	0.7(0.3-1.6)	reference dose<3.8- adjusted: year of birth, gender, body mass index, smoking, alcohol, total calories, vitamin A, folate, riboflavin, zinc, protein, carbohydrate
		28	99			5.7-<8.3	1.7(0.7-4.1)	
		39	100			8.3+	2.2(0.9-5.7)	
		31	99	Stomach_Distal_Adenocarcinoma		3.8-<5.7	1.6(0.8-3.2)	
		25	99			5.7-<8.3	1.8(0.8-3.8)	
		29	100			8.3+	1.6(0.7-3.7)	
	Ward, M. H._2008_ M/F_USA_Plant sources	28	102	Esophagus__Adenocarcinoma	Nitrite_ mg/day	0.36-<0.52	0.52(0.5-2.3)	reference dose<0.36- adjusted: year of birth, gender, body mass index, smoking, alcohol, total calories, vitamin A ,folate, riboflavin, zinc, protein, carbohydrate
		17	101			0.52-<0.67	0.6(0.2-1.3)	
		30	100			0.67+	1(0.4-2.4)	
		22	102	Stomach_Distal_Adenocarcinoma		0.36-<0.52	1.1(0.4-2.7)	
		29	101			0.52-<0.67	0.8(0.3-2.2)	
		30	100			0.67+	1.1(0.3-3.4)	
		27	99		Nitrate_	16.9-<26.2	0.9(0.5-1.8)	

		18	99	Esophagus__Adenocarcinoma	mg/day	16.2-<38.8	0.6(0.3-1.3)	reference dose<16.9- adjusted: year of birth, gender, body mass index, smoking, alcohol, total calories, vitamin A, folate, riboflavin, zinc, protein, carbohydrate
		24	100			>38.8	0.8(0.3-1.8)	
		28	99	Stomach_Distal_Adenocarcinoma		16.9-<26.2	1.2(0.6-2.5)	
		26	99			26.2-<38.8	1.4(0.7-2.9)	
		26	100			>38.8	1.6(0.7-3.6)	
	Ward, M. H._2008_M/F_USA_Water	12	55	Stomach_ distal_Adenocarcinoma	Nitrate_ mg/l	10	1(0.5-2.0)	reference dose= 0 years- adjusted: year of birth, gender, education, smoking, alcohol
		13	48			10	1.1(0.5-2.3)	
		12	55	Esophagus__ Adenocarcinoma		>10	0.8(0.4-1.8)	reference dose<10- adjusted: year of birth, gender, body mass index, smoking, alcohol
		15	48			>10	0.9(0.4-1.9)	
**25**	Zhang, T._2018_ M/F__Food	23	27	Esophagus	Nitrite_ mg/day	3.73 -7.13	1.175(0.53-2.59)	reference dose: 0 - 3.7350 - adjusted: sex and age
	Zhang, T._2018_ M/F__Food	25	25	Esophagus	Nitrite_ mg/day	7.13-17.32	1.373(0.61-3.05)	reference dose: 0 - 3.7350 - adjusted: sex and age
	Zhang, T._2018_ M/F__Food	31	19	Esophagus	Nitrite_ mg/day	17.32-71.57	2.256(1.01-5.02)	reference dose: 0 - 3.7350 - adjusted: sex and age
**26**	Zheng, J._2019_ M/F_USA_Animal sources	234	234	Pancrase_	NDMA_	.33	0.97(0.74-1.28)	reference dose:Q1=0.18- adjusted: age, energy, red and processed meet intake, sex, race, education level, BMI, alcohol level, history of diabetic, smoking, family history of pancreatic cancer
		201	235			.43	0.87(0.65-1.15)	
		272	234			.74	1.17(0.89-1.54)	
		196	234		Nitrate_	2.46	0.68(0.52-0.90)	reference dose:Q1=1.63- adjusted: age, energy, red and processed meet intake, sex, race, education level, BMI, alcohol level, history of diabetic, smoking, family history of pancreatic cancer
		220	235			3.05	0.74(0.56-0.97)	
		245	234			4.24	0.82(0.62-1.08)	
	Zheng, J._2019_ M/F_USA_Food	121	234		NDMA_	.45	0.99(0.75-1.31)	reference dose:Q1=0.28- adjusted: age, energy, red and processed meet intake, sex, race, education level, BMI, alcohol level, history of diabetic, smoking, family history of pancreatic cancer
		168	235			.58	1.03(0.78-1.37)	
		249	234			.99	1.03(0.78-1.37)	
		226	234		Nitrite_	.7	0.83(0.63-1.09)	reference dose:Q1=0.37- adjusted: age, energy, red and processed meet intake, sex, race, education level, BMI, alcohol level, history of diabetic, smoking, family history of pancreatic cancer
		225	235			.99	0.79(0.60-1.04)	
		215	234			1.55	0.68(0.51-0.91)	
		236	234		Nitrate_	34.37	0.93(0.71-1.23)	reference dose:Q1=21.67- adjusted: age, energy, red and processed meet intake, sex, race, education level, BMI, alcohol level, history of diabetic, smoking, family history of pancreatic cancer
		192	235			45.54	0.72(0.55-0.96)	
		271	234			73.29000000000001	1.07(0.81-1.41)	

Zheng, J._2019_ M/F_USA_Plant sources	227	234		NDMA_	.06	1.48(1.10-1.99)	reference dose:Q1=0.04- adjusted: age, energy, red and processed meet intake, sex, race, education level, BMI, alcohol level, history of diabetic, smoking, family history of pancreatic cancer
	300	235			.08	1.97(1.48-2.64)	
	272	234			.12	1.93(1.42-2.61)	
	230	234		Nitrate_	31.43	0.95(0.72-1.25)	reference dose:Q1=18.97- adjusted: age, energy, red and processed meet intake, sex, race, education level, BMI, alcohol level, history of diabetic, smoking, family history of pancreatic cancer
	204	235			42.58	0.8(0.61-1.07)	
	267	234			70.59	1.05(0.79-1.39)	
**27** Zhu, Y._2014_ M/F_Canada_Food	335	502	Colorectal_	NDMA_ micro/day	.07	1.06(0.83-1.37)	reference dose: 0.03- adjusted: age, sex, energy intake, BMI, smoking, alcohol, physical activity, education, income, NSAID, folate, supplement, province of residence
	354	493			.2	1.13(0.87-1.47)	
	336	513			.77	1.22(0.92-1.63)	
	407	441			2.29	1.42(1.03-1.96)	
	138	510	Colon_ Proximal_		.07	0.96(0.72-1.28)	
	123	488			.2	1.08(0.79-1.46)	
	132	519			.77	1.11(0.80-1.67)	
	129	446			2.29	1.58(0.80-1.67)	
	117	510	Rectum__		.07	1.06(0.77-1.45)	
	126	488			.2	1.19(0.86-1.66)	
	145	519			.77	1.15(0.81-1.63)	
	141	446			2.29	1.61(1.11-2.35)	
	117	510	Colon_ distal_		.07	1.06(0.77-1.45)	
	131	488			.2	1.24(0.89-1.72)	
	101	519			.77	0.97(0.68-1.39)	
	128	446			2.29	1.37(0.93-2.01)	
	117	496	Rectum__	Nitrite_ mg/day	.89	1.26(0.91-1.73)	reference dose:0.65- adjusted: age, sex, energy intake, BMI, smoking, alcohol, physical activity, education, income, NSAID, folate, supplement, province of residence
	126	520			1.12	1.2(0.84-1.71)	
	145	474			1.4	1.51(1.02-2.22)	
	141	455			1.92	1.45(0.94-2.24)	
	335	496	Colorectal_		.89	1.07(0.83-1.38)	
	354	520			1.12	0.99(0.75-1.30)	
	336	479			1.4	1.05(0.77-1.43)	
	407	450			1.92	1.09(0.77-1.54)	
	138	496	Colon_ Proximal_		.89	1.15(0.86-1.54)	
	123	520			1.12	0.91(0.66-1.26)	
	132	474			1.4	0.81(0.56-1.18)	
	129	455			1.92	0.95(0.63-1.43)	
	117	496	Colon_ distal_		.89	0.97(0.70-1.34)	
	131	520			1.12	0.93(0.65-1.32)	
	101	474			1.4	1.21(0.82-1.78)	

	128	455			1.92	1.32(0.85-2.04)	
	335	477	Colorectal_	Nitrate_ mg/day	91.45	1.27(0.99-1.60)	reference dose: 56.94- adjusted: age, sex, energy intake, BMI, smoking, alcohol, physical activity, education, income, NSAID, folate, supplement, province of residence
	354	488			124.81	1.19(0.93-1.52)	
	336	481			169.59	1.17(0.91-1.51)	
	407	518			264.14	0.89(0.68-1.16)	
	117	480	Rectum_		91.45	1.12(0.83-1.53)	
	126	489			124.81	1.23(0.90-1.69)	
	145	479			169.59	1.34(0.96-1.85)	
	141	516			264.14	1.03(0.73-1.46)	
	138	480	Colon_ Proximal_		91.45	1.25(0.93-1.66)	
	123	489			124.81	0.9(0.66-1.23)	
	132	479			169.59	1.06(0.78-1.46)	
	129	516			264.14	0.75(0.54-1.05)	
	117	480	Colon_ distal_		91.45	1.07(0.78-1.48)	
	131	489			124.81	1.24(0.90-1.71)	
	101	479			169.59	1.31(0.94-1.83)	
	128	516			264.14	1.01(0.71-1.45)	
